# Clinical, laboratory and molecular signs of immunodeficiency in patients with partial oculo-cutaneous albinism

**DOI:** 10.1186/1750-1172-8-168

**Published:** 2013-10-17

**Authors:** Laura Dotta, Silvia Parolini, Alberto Prandini, Giovanna Tabellini, Maddalena Antolini, Stephen F Kingsmore, Raffaele Badolato

**Affiliations:** 1 Department of Experimental and Clinical Sciences, Institute of Molecular Medicine “Angelo Nocivelli”, University of Brescia, Brescia, Italy; 2 Department of Molecular and Translational Medicine, University of Brescia, Brescia 25123, Italy; 3 Center for Pediatric Genomic Medicine, Children’s Mercy Hospital, Kansas City, MO 64108, USA; 4 Istituto di Medicina Molecolare “Angelo Nocivelli”, Universita' di Brescia, c/o Spedali Civili, Brescia 25123, Italy

**Keywords:** Primary immunodeficiency, Natural killer cells, Hemophagocytosis, Partial albinism

## Abstract

Hypopigmentation disorders that are associated with immunodeficiency feature both partial albinism of hair, skin and eyes together with leukocyte defects. These disorders include Chediak Higashi (CHS), Griscelli (GS), Hermansky-Pudlak (HPS) and MAPBP-interacting protein deficiency syndromes. These are heterogeneous autosomal recessive conditions in which the causal genes encode proteins with specific roles in the biogenesis, function and trafficking of secretory lysosomes. In certain specialized cells, these organelles serve as a storage compartment. Impaired secretion of specific effector proteins from that intracellular compartment affects biological activities. In particular, these intracellular granules are essential constituents of melanocytes, platelets, granulocytes, cytotoxic T lymphocytes (CTLs) and natural killer (NK) cells. Thus, abnormalities affect pigmentation, primary hemostasis, blood cell counts and lymphocyte cytotoxic activity against microbial pathogens. Among eight genetically distinct types of HPS, only type 2 is characterized by immunodeficiency. Recently, a new subtype, HPS9, was defined in patients presenting with immunodeficiency and oculocutaneous albinism, associated with mutations in the pallidin-encoding gene, *PLDN*.

Hypopigmentation together with recurrent childhood bacterial or viral infections suggests syndromic albinism. T and NK cell cytotoxicity are generally impaired in patients with these disorders. Specific clinical and biochemical phenotypes can allow differential diagnoses among these disorders before molecular testing. Ocular symptoms, including nystagmus, that are usually evident at birth, are common in patients with HPS2 or CHS. Albinism with short stature is unique to MAPBP-interacting protein (MAPBPIP) deficiency, while hemophagocytic lymphohistiocytosis (HLH) mainly suggests a diagnosis of CHS or GS type 2 (GS2). Neurological disease is a long-term complication of CHS, but is uncommon in other syndromic albinism. Chronic neutropenia is a feature of HPS2 and MAPBPIP-deficiency syndrome, whereas it is usually transient in CHS and GS2. In every patient, an accurate diagnosis is required for prompt and appropriate treatment, particularly in patients who develop HLH or in whom bone marrow transplant is required. This review describes the molecular and pathogenetic mechanisms of these diseases, focusing on clinical and biochemical aspects that allow early differential diagnosis.

## Introduction

Hypopigmentation syndromes represent a readily distinguished group of diseases. Pigment dilution may involve skin, hair and iris, and generally is manifest at birth. Genetic defects of melanin biosynthesis are inherited as autosomal recessive Oculocutaneous Albinism (OCA) or X-linked Ocular Albinism (OA), in which abnormal pigmentation is an isolated manifestation [1]. However, hypopigmentation may also represent a feature of genetic disorders characterized by immunodeficiency, including Chediak Higashi Syndrome (CHS), Griscelli Syndrome (GS), Hermansky-Pudlak type 2 Syndrome (HPS2) and MAPBPIP-deficiency Syndrome. Recently a new form of HPS-like syndrome, known as HSP-9, has been added to the causes of immunodeficiency-associated albinism.

All causal genes that have been linked to albinism appear to play a role in the biogenesis, function or trafficking of intracellular organelles, particularly of secretory lysosomes [2]. Cell types containing secretory lysosomes include melanocytes, hematopoietic cells and renal tubular cells. In melanocytes, secretory lysosomes are known as melanosomes, and are involved in melanin pigment secretion. Among cells of hematopoietic lineage, the cytotoxicity of T and Natural Killer (NK) cells requires tightly regulated secretion of cytolytic enzymes stored in secretory lysosomes. In addition, antigen presentation to T cells by macrophages, dendritic cells and B cells involves MHC-class-II positive secretory lysosomes for proper transport and processing of endocytic antigens [2]. Finally, primary hemostasis is mediated by platelet clotting agents, stored in secretory lysosomes, while, neutrophil antimicrobial activity depends upon regulated release of cytosolic granules.

Defects in proteins involved in the function of secretory lysosomes affect biological activity of these cells leading to hypopigmentation and various degrees of immunodeficiency [3,4]. A thorough evaluation of a patient presenting with partial albinism and immunodeficiency will feature clinical, laboratory and molecular aspects aiming to a prompt diagnosis and effective treatment.

### Disease name

Partial Oculocutaneous Albinism and Immunodeficiency (OCA-ID).

### Definition

Partial Oculocutaneous Albinism and Immunodeficiency (OCA-ID) is a group of five autosomal recessive syndromes clinically characterized by hypopigmentation of skin, hair and eyes, associated with recurrent infections. While these diseases have similar cutaneous and ocular manifestations, including partial albinism, nystagmus and strabismus, the hematologic symptoms can be extremely heterogeneous and vary from mild bleeding, as seen in HPS2 patients, to hemophagocytic syndrome, as observed in CHS and GS2 patients. All patients with OCA-ID display a moderate to high susceptibility to both viral and bacterial infections that can manifest variously as delayed clearance of herpes viruses, and often by respiratory and cutaneous bacterial infections. Defective cytotoxicity of both NK and cytotoxic T cells (CTL) is observed in all patients with OCA-ID, but to a variable extent, being more severe in patients with CHS and GS2. Neutropenia is a chronic feature in HPS2 and in MAPBPIP-deficiency, but can be transiently observed in the other conditions [Table 1].

**Table 1:** Primary Immunodeficiencies that associated with oculocutaneous albinism

	**Locus**	**Oculocutaneous albinism**	**Bleeding disorders**	**Short stature**	**Neurological symptoms**	**HLH**	**Neutropenia**	**NKs defects**	**CTLs defects**	**Giant granules**
CHS	CHS1	+	+	-	+	+	+/-*	+	+	+
GS2	RAB27A	+	-	-	-	+	+/-*	+	+	-
HPS2	ADTB3A	+	+	-	-	+	+	+	+	-
HPS9	PLDN	+	-	-	-	-	-	+	N/A	-
MAPBPIP deficiency	LAMTOR2	+	-	+	-	-	+	+	+	-

*Transient neutropenia can be observed.

CHS Chediak-Higashi syndrome; GS2 Griscelli syndrome type 2; HPS-2 Hermanski-Pudlak syndrome-type 2; HLH hemophagocytic lymphohistiocytosis.

### Epidemiology

Partial Albinism and Immunodeficiency disorders are extremely rare. Each of them has a prevalence of < 1/1,000,000 without crossover-enrichment in selected populations.

### Patients presenting with oculo cutaneous albinism and hemophagocytic lymphohistiocytosis

#### Hemophagocytic lymphohistiocytosis (HLH)

HLH represents a life-threatening condition commonly affecting about 50-85% of CHS patients within the first decade and, in the case of the majority of GS2 patients, frequently in the first year of life [5,6]. A single case of HLH has been reported in one patient with HPS2.

HLH is a severe hyperinflammatory disease caused by uncontrolled but ineffective immune response. It is also known as “accelerated phase”: it manifests as prolonged fever, lymphadenopathy, hepatosplenomegaly, signs of liver dysfunction (including jaundice, elevated transaminases, hypofibrinogenemia and/or hypertriglyceridemia, high level of ferritin and lactate dehydrogenase), cytopenia of at least two hematopoietic cell lineages (commonly neutropenia, anemia, thrombocytopenia or pancytopenia) [7]. Neurological manifestations, ranging from cranial nerve palsy to seizures and decreased level of consciousness, may occur. Cerebrospinal fluid shows pleocytosis, increased proteins, or both [8]. Histopathology reveals lymphoproliferative infiltration of bone marrow and reticuloendothelial system. The cause is a defective cytotoxic activity leading to impaired down regulation of immune response and sustained activation and proliferation of CTL and NK cells [9]. These cells produce large amounts of cytokines (such as IFNy, TNFalpha, GM-CSF) activating macrophages and dendritic cells. The latter migrate to sites of inflammation, thus infiltrating tissues and organs and producing high levels of proinflammatory cytokines and chemokines that are responsible for the laboratory signs and symptoms of HLH. Therefore, diagnosis is based on clinical and laboratory criteria, according to the guidelines of the Hystiocyte Society lastly revised in 2007. For the diagnosis of HLH, five out of eight criteria are required; they are: prolonged fever, enlarged spleen, low or absent NK cell function, abnormalities in two or more blood cell lineages, increased triglycerides or reduced fibrinogen, increased serum ferritin, evidence of hemophagocytosis in bone marrow but not malignancy, abnormally high soluble CD25 in blood as sign of T-cell activation.

Moreover, HLH may occur during the course of Epstein-Barr virus infections, when it may resemble lymphoma [10]. In patients who present with HLH as the first manifestation of disease and with mild or absent symptoms of OCA-ID, other inherited forms of HLH may be considered. Familial hemophagocytic lymphohistiocytosis is also genetically heterogeneous. There are five genetically distinct types which share an autosomal recessive pattern of inheritance. The causal genes are known for four types, and belong to the cytolytic granule-dependent exocytosis pathway. They are perforin (FHL2), *UNC13D* (FHL3), syntaxin-11 (FHL4), syntaxin-binding protein-2 (FHL5). In these cases, HLH represents the primary and only manifestation of immunodeficiency [11]. If untreated, HLH may be fatal within a few weeks. HSCT is strongly indicated as the only curative treatment.

### Chediak-higashi syndrome

CHS is an autosomal recessive disorder characterized by partial albinism associated with immune dysfunction, bleeding diathesis and progressive neurologic deterioration. The risk of hemophagocytic lymphohistiocytosis is estimated at 85% [5,12-14].

#### Clinical phenotype

*Partial Albinism*. Patients present a variable degree of hypopigmentation affecting skin, hair and eyes [5,14]. Skin color can vary from milky-white to slate gray, and hypopigmentation is often appreciated only by comparison with other family members. Skin hypopigmentation is associated with increased risk of sun damage and skin cancer; uncommonly, CHS may present with hyperpigmentation, leading to suspicion of other photosensitivity diseases characterized by hyperpigmentation with consequent delay in diagnosis [15]. Hair color may appear blonde to light brown, often with a distinguished silvery or metallic sheen. Iris hypopigmentation may be associated with decreased retinal pigmentation, nystagmus and impaired visual acuity.

*Infections*. The clinical history in CHS is typically remarkable for recurrent and severe bacterial infections since childhood [14]. Skin and respiratory tract are mainly involved; *Staphylococcus* and *Streptococcus* are the species most frequently isolated. Viral and fungal infections, however, have also been described. According to recent reports, severe periodontitis may also be an indication to raise suspicion of CHS [16].

*Bleeding diathesis*. Patients present a prolonged bleeding time and may manifest mildly disordered platelet aggregation, including epistaxis, gum/mucosal bleeding, petechiae, and easy bruising, that do not usually require any treatment [5,14].

*Neurological manifestations*. Patients who survive into early adulthood may develop motor and sensory neuropathies, balance abnormalities, ataxia, tremor, absent deep-tendon reflexes, and low cognitive abilities. In addition, neurologic symptoms can also be observed in older patients presenting with an atypical, milder form of CHS, manifesting with dementia, parkinsonism, peripheral neuropathy [5,14].

#### Molecular genetics and mechanisms

The human gene identified as causal for CHS, *LYST/CHS1,* is located at chromosome 1q42.1-q42.2 [17-19]. LYST (lysosomal trafficking regulator) is a cytosolic protein of approximately 430 kDa which is highly conserved through evolution and is expressed at low levels in all cell types [20]. Its precise function remains unknown but it is hypothesized its role in regulating lysosome size and trafficking, particularly by regulating membrane fission/fusion events [21,22]. The pathognomonic feature of CHS is the presence of giant lysosomes and lysosome-related organelles in all cell types [23,24]. While the degradative function of lysosomes might be conserved, the exocytic pathway of secretory lysosomes is impaired in leukocytes of CHS patients. All mutations identified to date are missense or nonsense substitutions, small coding deletions or insertions that often result in protein truncation or nonsense mediated decay. A correlation between specific genotypes and clinical phenotypes has been hypothesized: loss-of-function mutations are associated with severe, childhood-onset forms, while missense mutations occur in milder adolescent- or adult-onset forms of the disease [25,26]. In addition, other studies suggest a relation between the extent of the cellular defect and the clinical phenotype [27]. Recently two novel heterogeneous mutations have been discovered, but their relationship with the clinical phenotype remains unclear [28]. Each clinical manifestation of CHS is associated with a defect of a specific cell type and to the formation of secretory enlarged lysosomes in these cells [29-34]. Bleeding diathesis is related to the decreased pool of platelet dense granules that is needed for a normal aggregation response, while antimicrobial activity defect is due to the reduced amount of neutrophil enzymes, as well as the impaired exocytosis of lytic proteins is responsible for the impairment of NK and CTL cell cytotoxicity. Pigment dilution is related to the defective migration of giant melanosomes from the melanocyte dendrites to surrounding keratinocytes. Therefore, the presence of giant organelles may affect neutrophil chemotaxis and degranulation as well as giant inclusions. In neuronal cells, formation of giant granules might have a role in the pathogenesis of the neurological manifestations of CHS patients.

#### Management

Episodes of infection, clinical and ophthalmological findings may suggest the diagnosis of CHS. Clinical diagnosis is firstly supported by the presence of peroxidase-positive giant inclusions in white blood cells, by detection of pigment clumping in the light microscopy analysis of hair and eventually by studies revealing abnormal platelet aggregation [14]. Electron microscopy can demonstrate reduced number and irregular morphology of platelet dense-bodies [29,30]. Of note, giant granules resembling those seen in CHS may be revealed in acute and chronic myeloid leukemia. NK cell counts are generally normal but cytotoxic activity is impaired [33]. Neutropenia may coexist with abnormal functioning (typically chemotaxis and intracellular bactericidal activity) [32]. Immunoglobulin levels, complement are generally normal while delayed hypersensitivity may be impaired. Definitive diagnosis is based on molecular genetic testing of *LYST*. Of note, cases of neurological involvement, subtle albinism, bleeding manifestations, even without history for recurrent infections, may be considered for atypical and mild phenotypes of CHS [5]. Prognosis is poor because death frequently occurs in the first decade of life due to infections or development of HLH. Treatment of infections requires prompt and aggressive antimicrobial therapies; while prophylaxis should be considered according to the frequency of infectious episodes. A prompt diagnosis of CHS can prevent the development of HLH by initiating appropriate treatments. The most effective treatment for the hematological and immune defects of CHS is HSCT, albeit there is no evidence of efficacy in delaying or preventing progressive neurological dysfunction [35,36].

### Griscelli syndrome type 2

Griscelli syndrome is a rare autosomal recessive disorder first described in 1978 as partial albinism associated with immunodeficiency [37]. Type 2 Griscelli syndrome can be distinguished from the other two forms of this disorder on the basis of distinctive clinical and molecular features. Griscelli syndrome type 1 (GS1) features primary neurological disease and pigment anomalies and it is caused by mutations in the *MYO5A* gene encoding the motor protein myosin-Va [38]. The clinical features of type 3 (GS3) are restricted to hypopigmentation of skin, and hair. GS3 is caused by mutations in the melanophilin gene *MLPH*[39]. Among GS subtypes, only patients with type 2 commonly may develop HLH.

#### Clinical phenotype

Children with GS2 frequently present with silver-gray hair and relatively light skin color. Usually they have an increased susceptibility to recurrent pyogenic infections and may present with recurrent episodes of fever, hepatosplenomegaly and lymphadenopathy [40]. In the majority of GS2 patients, the development of HLH, known also as the “accelerated phase”, occurs from 6 to 12 months of age [6]. While neurological symptoms can be observed in GS2 patients, they are related to the development of HLH in these subjects, as the gene *RAB27A* associated with GS2 is not expressed in neuronal cells.

#### Molecular genetics and mechanisms

GS2 is caused by mutations in the *RAB27A* gene, which maps to chromosome 15q21 and encodes a small GTPase that regulates vesicular fusion and trafficking. In particular Rab27a is required for peripheral anchorage of melanosomes in melanocytes, as well as exocytosis of cytolytic granules in CTL and NK cells. In addition, Rab27a is crucial for docking of cytolytic granules to the plasma membrane secondary to target-cell recognition and TCR signal activation [6,41,42].

#### Management

Most commonly the diagnosis of GS2 occurs between 4 months and 7 years [40]. Parental consanguinity and a history of familial deaths may be suggestive. Clinical suspicion of GS2 can be supported by light microscopic examination of hair shafts, typically showing irregular large clumps of melanin pigment. Electron microscopic evaluation of skin reveals numerous mature melanosomes in melanocytes with few melanosomes in adjacent keratinocytes [37,40]. Giant granules are absent in peripheral leukocytes. Defects of adaptive immunity are variable: laboratory tests may reveal defective NK cytoxicity together with an impaired delayed-type hypersensitivity response [41]. Commonly, however, granulocyte and lymphocyte count and functions are normal, while immunoglobulin levels may be normal, decreased or increased. As discussed below, since HLH typically develops in GS2, the disease is fatal without HSCT [43,44]. Immunosuppressive therapy is reported to improve patient symptoms as a palliative treatment or to induce remission until HSCT can be performed [45]. There is evidence of rescue of CTL activity using a retroviral vector to mediate the transfer of RAB27A gene, opening an alternative possibility for GS2 treatment [46]. A prompt initiation of an appropriate treatment can prevent complications, including the neurological sequelae of the disease.

## Patients presenting with oculocutaneous albinism and neutropenia

### Hermansky pudlak syndrome type 2 (HPS2)

HPS2 belongs to a genetically heterogenous group of autosomal recessive disorders that share oculocutaneous albinism and platelet storage disease. In humans, nine causative genes have been cloned and sequenced: each defective gene is involved in formation, transport or fusion of intracellular vesicles of lysosomal lineage [47]. Type 2 represents the HPS type that was first associated to immunodeficiency, specifically neutropenia.

#### Clinical phenotype

Clinical signs suggestive for HPS2 may be detectable at birth [48]: they include horizontal nystagmus, ocular hypopigmentation, including iris trans-illumination and hypopigmentated areas of fundus oculi. As a consequence, decreased visual acuity is frequent. Hair color ranges from white to dark brown. Hair may be sparse and white and may darken over time. In the same way, skin varies from white to brown and often must be compared to the skin tone of other family members to be distinguished. As with other types of albinism, patients are susceptible to sun damage and there is a higher risk for skin malignancies. In childhood, distinctive facial features may become evident, in particular, epicanthal folds, posteriorly rotated ears, broad nasal root and retrognathia. HPS2 patients may have a prolonged bleeding time (up to > 15 minutes) and manifest spontaneous soft tissue bruising and mucosal bleeding, while major hemorrhages into joints, brain or other organs are rare. Typically the first sign of a bleeding diathesis in HPS patients consists of excess bruising beginning at the time of first ambulation. Otherwise, episodes of epistaxis are frequent but often remit during adolescence. Other events that may result in excessive bleeding include dental extractions, surgeries, acute colitis, menstrual periods, and childbirth. Recurrent infections are a prominent feature of HPS2 [49]. Bacterial infections mainly include upper respiratory infections, otitis media and pneumonias. Moreover, HPS2 patients display higher susceptibility to viral infections and certain malignancies, such as Hodgkin’s lymphoma [50]. The defects of cytotoxic activity that have been reported in HPS2 patients might suggest an increased risk of HLH. However, only one case of HLH in HPS2 has been described in literature. That patient was also heterozygous for a RAB27a mutation [51], suggesting that other genetic or environmental factors probably contributed to HLH development in this patient.

#### Molecular genetics and mechanisms

HPS2 is caused by mutations in *AP3B1* gene, on chromosome 5q14.1, encoding the β3A subunit of the heterotetrameric adapter protein (AP-) 3 complex [49,52]. Defects in β3A subunit prevent the formation of the entire AP-3 complex. In mammalian cells there are 4 types of adaptor protein complexes that are involved in cellular protein trafficking. In particular, AP-3 directs post-translational trafficking of intraluminal cargo proteins from the trans-Golgi network to lysosomes [53-55]. The absence of the AP-3 complex impairs trafficking of lysosome-targeted proteins to and from lysosomes so that proteins trafficking to lysosomes will accumulate at the plasma membrane. Consequently, regulation and functioning of specific cells related to secretory lysosomes are defective [56,57].

In melanosomes, there is evidence that AP-3 regulates tyrosinase trafficking, which is essential for the synthesis of melanin. In platelets, the absence of dense granules, that contain ADP, ATP, serotonin, calcium and polyphosphates, which are required for platelet aggregation, is a typical feature of the disease.

In neutrophils, AP-3 mediates trafficking of neutrophil elastase to lysosome-like granules, known as azurophilic granules. Neutrophil elastase is required for normal differentiation of myeloid progenitor cells to mature neutrophils. Abnormal trafficking of neutrophil elastase is associated with neutropenia, although the precise mechanism remains to be defined [58]. Thus, HPS2 patients exhibit arrested maturation of neutrophil precursors at the stage of pro-myelocytes. The neutrophil count in HPS2 patients usually increases in response to infection or to Granulocyte-Colony Stimulating Factor (G-CSF) therapy [57,58].

In CTLs in HPS2, defects in lytic granule trafficking and functioning are associated with impaired cytotoxicity, particularly due to failure to secrete lysosomal lytic enzymes, such as perforin and granzymes, in response to receptor signaling [56,57]. Moreover, NK cells have reduced lysosomal pools of perforin, while granzyme levels are normal, suggesting possible defects of NK cell differentiation.

In addition, there is evidence for a role of the AP-3 complex in antigen presentation; notably AP-3 deficiency results in impaired antigen presentation by CD1b and impaired microbial lipid antigen presentation to T-cells [59,60]. CD1b binding to AP-3 is required for proper localization in the MHC class II compartment, for appropriate sorting from the lysosome to the plasma membrane and for antigen presentation. In particular, there is a subset of T cells, known as invariant NK T-cells (iNKT) expressing a semi-invariant TCR, which confers the capacity to recognize a limited number of glycosphingolipids presented by APCs in the context of CD1b or of the murine ortholog CD1d [61,62]. As consequence, these cells can play a stimulatory role toward other immune cells or can exert immune regulatory function. Lack of AP-3 might deregulate this signaling and activation pathway contributing to NK-T cell deficiency and to susceptibility to bacterial and viral infections [60,62].

#### Diagnosis and management

Laboratory tests reveal neutropenia in HPS2 patients. Platelet count is normal or increased but platelet aggregation response is altered, generally with an abnormal response to collagen and adenosine diphosphate, but normal response to ristocetin. Electron microscopy of platelets reveals reduction of dense bodies. Lymphocyte subpopulations and proliferative response to mitogens are generally normal. NK cells and CTL cells are normal in number or slightly reduced with an impaired cytolytic activity.

Molecular analysis of *AP3B1* allows definitive diagnosis. A careful follow up is necessary to monitor when G-CSF treatment is required.

### Hermansky pudlak syndrome type 9: pallidin deficiency

HPS9 is the most recently defined subtype of Hermansky-Pudlak syndrome. It was recently described in patients presenting with clinical manifestations suggestive of HPS, but with negative molecular tests for previously known HPS types [63]. The molecular basis of HPS9 in one of the two pedigrees described was identified by exome sequencing [64].

#### Clinical phenotype

The two cases reported to date in the literature shared partial albinism, nystagmus, normal neurological development and absence of platelet delta granules, but lacked the bleeding manifestations typical of HPS2 [63,64]. One case had recurrent infections, especially of the skin, and transient leukopenia [64].

#### Molecular genetics and mechanisms

The two patients had the same homozygous mutation, c.232C>T (p.Q78X), in exon 3 of the pallidin gene (*PLDN*, chr15:45895305C>T), which led to undetectable expression of the protein product in NK cells. The full length *PLDN* transcript, which contains exon 3, is ubiquitously expressed in adult and fetal tissues with the exception of brain. A second *PLDN* transcript, that has a strong expression in the brain, arises as a result of alternative splicing and skipping of exon 3, which contains the mutation observed in the two HPS9 patients [63]. Pallidin is a subunit of the protein complex BLOC-1 (biogenesis of lysosome-related organelles complex-1). To date, mutations have been described in three human BLOC-1 genes and in five mouse BLOC-1 genes. In melanocytes, the absence of pallidin may impair the stability and functioning of other BLOC-1 proteins such as syntaxin-13, which is an endosome t-SNARE (target-SNAP -Soluble N-ethylmaleimide-sensitive factor Attachment Protein- Receptor), and may alter intracellular trafficking of TYRP1, a protein which is crucial for melanosome maturation and melanin production [63]. In NK cells, pallidin regulates the expression of lysosomal membrane proteins, particularly CD107a and CD63, akin to HPS2. It has been shown that pallidin absence results in impairment of degranulation and cytolytic activity of NK cells thus leading to immune defects [64].

#### Management

HPS9 may be suspected together with HPS2 in patients with albinism and recurrent infections; although the two HPS9 patients did not present bleeding manifestations, further cases are needed to more fully define the clinical phenotype. Antibiotic prophylaxis can be used in patients with recurrent infections.

### Deficiency of endosomal adaptor protein MAPBPIP

A recently defined primary immunodeficiency syndrome features congenital neutropenia and B-cell and CTL deficiency together with partial albinism and short stature [65].

#### Clinical phenotype

The reports to date in the literature describe children with recurrent bronchopulmonary infections, mainly by *Streptococcus pneumonia,* with the short stature as an additional pathognomonic feature [65]. The extent of albinism is variable.

#### Molecular genetics and mechanisms

MAPBPIP, is encoded by the *LAMTOR2* gene, is an ubiquitous endosomal protein (also known as p14). It is localized on the outer membrane of late endosome, and functions as a scaffold molecule involved in mitogen-activated protein kinase (MAPK) signaling [66,67]. MAPBPIP plays an essential role in many cellular functions such as subcellular compartmentalization of signals and cellular responses to cell surface receptor signaling, as evidenced by early lethality in *Lamtor2* knock-out mice [65]. A homozygous point mutation in the 3’UTR of *LAMTOR2* located on chromosome 1q21 causes decreased *LAMTOR2* mRNA expression and consequently decreased protein levels. Aberrant lysosome function due to MAPBPIP deficiency affects neutrophils, B cells, CTLs and melanocytes. Neutrophils show an altered ultrastructure of azurophilic granules and decreased microbiocidal activity in phagosomes. In MAPBPIP-deficient cells, cytokine-receptor-mediated ERK phosphorylation is defective and a marked delocalization of late endosomes can be observed.

#### Management

Recurrent bacterial infections and growth delay in a patient affected by severe peripheral neutropenia may be suggestive for this new form of OCA-ID. Neutrophil maturation in bone marrow is supposed to be preserved. To date, in MAPBPIP deficiency syndrome immunological tests reveal decreased CTL cytotoxicity, and increased number of naïve B cells but a reduced number of memory B cells [65]. Definitive diagnosis depends on molecular analysis demonstrating mutations in *MAPBPIP* gene. Treatment with G-CSF is required to reduce frequency and severity of infections.

### Differential diagnosis

Hypopigmentation of skin, hair and eyes can be easily observed at birth in the majority of patients with oculocutaneous albinism, while the degree of hypopigmentation is variable, often according to parent’s pigmentation. Depigmentation is commonly extensive in HPS2 and CHS patients and affects skin, coloring from white to gray, hair, tingling from blonde to light brown, or with a silvery or metallic sheen typical of CHS, and eyes appearing gray, blue or rarely brown [5,48]. In GS2, hypopigmentation affects skin and hair, which is typically silvery-gray [41], while in MAPBPIP deficiency albinism is variable and the pathognomonic sign is the short stature [65]. Ocular symptoms, particularly nystagmus, iris translucency and reduced retinal pigmentation, are similarly present in neonates, in patients with CHS or HPS2. Symptoms related to immunodeficiency usually become evident in childhood and therefore, at birth, the phenotype of OCA-ID disorders cannot be easily distinguished from other causes of albinism [1]. Similar ocular and cutaneous features can be found in patients with Hermansky-Pudlak syndromes (HPS1-8), but these patients may also have mild bleeding defects and, at later ages, interstitial pulmonary fibrosis. Other rare inherited disorders that should be excluded in patients with albinism are Waardenburg Syndrome type II (WS2), which is associated with sensor-neural deafness, and Griscelli syndrome type 1, which is also characterized by neurological disorders and by a unique distribution of pigment in hair. Although differential diagnosis of OCA-ID within these inherited disorders can be easy at later ages, after observation of repeated episodes of infections, the diagnosis should not be delayed if OCA-ID is suspected because invasive infections may be fatal in such patients. Moreover, neurological symptoms appear along time in CHS patients, being characterized mainly by peripheral neuropathy [14]. HLH occurs primarily in CHS and GS2 [5,6]. Only one case of HLH has been reported in HPS2 [51]. Given the limited number of cases, it is impossible to define the risk of HLH in patients with p14 or pallidin deficiency. HLH is a life threatening condition that may be prevented with the appropriate treatment when diagnosis is defined. Therefore, when one of these disorders is suspected, microscopic, immunological and genetic studies should be promptly performed.

In CHS, microscopic evaluation of hair shafts reveals regular small melanin aggregates, while in GS2 irregular large clumps of melanin pigment are characteristic. Moreover, microscopic evaluation of skin shows in CHS giant melanosomes both in keratinocytes and melanocytes; while in GS2, epidermal melanocytes are filled with numerous mature melanosomes and adjacent keratinocytes with only rare melanosomes. Evaluation of neutrophil organelle morphology and count is useful in CHS, HPS2 and MAPBPIP deficiency, but unrevealing in GS2. Neutropenia is chronic in HPS2 and MAPBPIP deficiency, while it can be transiently observed in CHS and GS2. Neutrophil functions, including bactericidal activity, chemotaxis and degranulation, are defective only in CHS, where microscopic examination of peripheral blood smear reveals giant inclusions that are typical of this disorder [24]. For prompt identification of OCA-ID, evaluation of NK or CTL cytotoxic activity against susceptible cells is important, because it is impaired in all these syndromes. Immunoglobulin levels are typically normal in HPS2 and CHS patients, while in GS2 immunoglobulin may be increased or reduced. In MAPBPIP deficiency, B-memory cells are reduced, with consistently decreased serum IgM levels [65]. In CHS and GS2, delayed hypersensitivity may be impaired, while no alterations are described in the other syndromes [14]. Functional platelet studies reveal defects in aggregation, while their count is normal or increased in HPS2 and CHS. Molecular genetic testing is the gold standard for definitive diagnosis. Newer approaches, such as genome, exome or gene panel sequencing, are starting to be employed for differential diagnosis of disorders such as these, where there is substantial genetic heterogeneity [68-76].

### Genetic counseling and antenatal diagnosis

All five types of OCA-ID share an autosomal recessive pattern of inheritance. Therefore, the parents of a child with any of these conditions are usually obligate carriers of the disease and are asymptomatic. The risk of recurrence in a family with an affected child is 25%. Although consanguinity may be suggestive, meanwhile, many cases are sporadic and family history may be negative.

Carrier detection and prenatal diagnosis are possible by molecular analysis of the causative gene after identification of the genetic mutation in the proband (affected individual). For prenatal diagnosis, genetic testing can be performed on DNA extracted from a chorionic villus sample (CVS) at 10–12 weeks gestation or cultured amniocytes. In principle, preimplantation diagnosis by molecular genetic analysis would be feasible following identification of the causative mutation(s) in the proband.

## Conclusion

**Figure 1:**
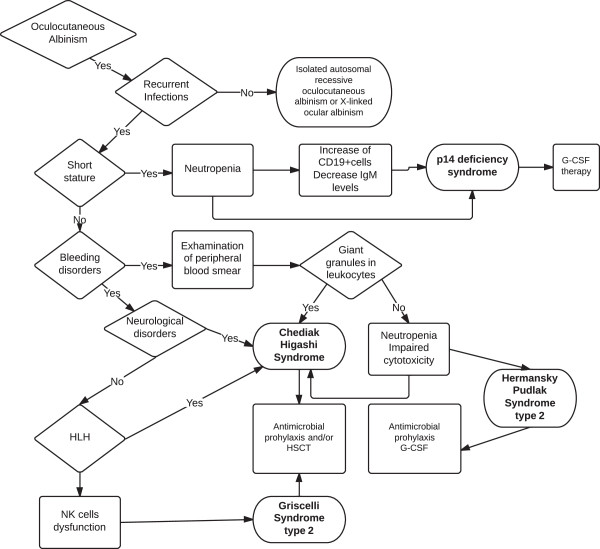
Diagnostic approach to patients with oculocutaneous albinism.

Differential diagnosis of Chediak-Higashi syndrome, Griscelli syndrome type 2, Hermansky-Pudlak syndrome type 2 and type 9 and MAPBPIP deficiency syndrome requires clinical, biochemical and molecular criteria. Clinical suspicion of OCA-ID should promptly lead to the performance of laboratory tests, including blood cell counting, blood smear analysis and immunoglobulin levels. In addition, clinical investigation of these patients should include assessment of NK or CTL cytotoxicity activity, light microscopic evaluation of hair and ophthalmological studies [Figure 1]. Finally, defining the causal genetic mutation may allow prompt institution of the appropriate treatments in affected patients and promote molecular tests in family members when necessary.

## Abbreviations

CHS: Chediak Higashi syndrome; GS: Griscelli syndrome; HPS: Hermansky-Pudlak syndrome; OCA: Oculocutaneous Albinism; OCA-ID: Oculocutaneous Albinism and Immunodeficiency; NK: Natural Killer; CTL: Cytotoxic T cells; HLH: Hemophagocytic lymphohistiocytosis; MHC II: Histocompatibility complex class II; HSCT: Hematopoietic Stem Cell Transplantation.

## Competing interests

The authors declare no conflict of interests.

## Authors’ contributions

All authors contributed to a draft of the manuscript and were subsequently involved in revising the manuscript critically for important intellectual content. All authors read and approved the final manuscript.
